# FLASH: a next-generation CRISPR diagnostic for multiplexed detection of antimicrobial resistance sequences

**DOI:** 10.1093/nar/gkz418

**Published:** 2019-05-22

**Authors:** Jenai Quan, Charles Langelier, Alison Kuchta, Joshua Batson, Noam Teyssier, Amy Lyden, Saharai Caldera, Aaron McGeever, Boris Dimitrov, Ryan King, Jordan Wilheim, Maxwell Murphy, Lara Pesce Ares, Katherine A Travisano, Rene Sit, Roberto Amato, Davis R Mumbengegwi, Jennifer L Smith, Adam Bennett, Roly Gosling, Peter M Mourani, Carolyn S Calfee, Norma F Neff, Eric D Chow, Peter S Kim, Bryan Greenhouse, Joseph L DeRisi, Emily D Crawford

**Affiliations:** 1 Chan Zuckerberg Biohub, San Francisco, CA 94158, USA; 2 Department of Biochemistry and Biophysics, University of California San Francisco, San Francisco, CA 94158, USA; 3 Division of Infectious Diseases, Department of Medicine, University of California San Francisco, San Francisco, CA 94158, USA; 4 Department of Pediatrics, University of California San Francisco, San Francisco, CA 94158, USA; 5 Chan Zuckerberg Initiative, Redwood City, CA 94063, USA; 6 Division of HIV, Infectious Diseases and Global Medicine, Department of Medicine, University of California San Francisco, San Francisco, CA 94143, USA; 7 Wellcome Sanger Institute, Hinxton CB10 1SA, UK; 8 Multidisciplinary Research Centre, University of Namibia, Windhoek 93Q5+48, Namibia; 9 Department of Epidemiology and Biostatistics, University of California San Francisco, San Francisco, CA 94158, USA; 10 Section of Critical Care Medicine, Department of Pediatrics, University of Colorado School of Medicine and Children's Hospital Colorado, Aurora, CO 80045, USA; 11 Division of Pulmonary and Critical Care Medicine, Department of Medicine, University of California San Francisco, San Francisco, CA 94158, USA; 12 Center for Advanced Technology, University of California San Francisco, San Francisco, CA 94158, USA; 13 Department of Biochemistry, Stanford University School of Medicine, Stanford, CA 94305, USA; 14 Stanford ChEM-H, Stanford, CA 94305, USA; 15 Department of Microbiology and Immunology, University of California San Francisco, San Francisco, CA 94158, USA

## Abstract

The growing prevalence of deadly microbes with resistance to previously life-saving drug therapies is a dire threat to human health. Detection of low abundance pathogen sequences remains a challenge for metagenomic Next Generation Sequencing (NGS). We introduce FLASH (Finding Low Abundance Sequences by Hybridization), a next-generation CRISPR/Cas9 diagnostic method that takes advantage of the efficiency, specificity and flexibility of Cas9 to enrich for a programmed set of sequences. FLASH-NGS achieves up to 5 orders of magnitude of enrichment and sub-attomolar gene detection with minimal background. We provide an open-source software tool (FLASHit) for guide RNA design. Here we applied it to detection of antimicrobial resistance genes in respiratory fluid and dried blood spots, but FLASH-NGS is applicable to all areas that rely on multiplex PCR.

## INTRODUCTION

Emerging drug resistant pathogens represent one of the most significant threats to human health. Drug resistant infections currently claim 700 000 lives per year and are predicted to cause 10 million deaths annually by 2050 (1). Antimicrobial susceptibility information is crucial to implement targeted and effective therapeutic interventions, but is often unobtainable due to the need to first isolate a pathogen in culture (2), a process that can require days to months depending on the organism, and has low success rates in the setting of prior antibiotic use (3,4). Ongoing and emerging drug resistance is a central challenge for malaria and other parasitic diseases as well (5,6). To limit the spread and impact of anti-malarial drug resistance, real-time surveillance of resistance patterns is essential. Current methods include *in vivo* efficacy studies from patient samples, *in vitro* phenotypic resistance assays and PCR-based detection of gene mutations associated with drug resistance (7). While rapid genotyping offers many advantages over organism viability studies, in areas of high disease transmission it is confounded by the presence of co-infections where a low-abundance strain may contain clinically and epidemiologically relevant sequences essential for assessing transmission patterns (8).

Metagenomic Next Generation Sequencing (mNGS) has proven invaluable for detecting pathogens in clinical samples (9–11); however, the key issue of antimicrobial resistance (AMR) detection is not easily addressed by mNGS alone. While interrogation of antibiotic resistance genes is readily achievable from cultured isolates, it is often not possible from direct clinical specimens due to low target abundance and high background derived from the host. Thus, detection of low abundance targets is a central challenge in clinical diagnostics, and a solution would have universal relevance across medical disciplines. Methods combining multiplex PCR with NGS, such as 16S rRNA gene profiling and AmpliSeq (12) provide effective enrichment but are hampered by cost, scalability and inflexibility when new targets are discovered. Other approaches that rely on probe-based hybrid capture suffer from high off-target rates, long incubation times, and expensive reagents.

The exquisite and programmable specificity of CRISPR systems has inspired many novel uses of their enzymes beyond genome engineering since the characterization of *Streptococcus pyogenes* Cas9 in 2012 (13). The SHERLOCK (14,15) and DETECTR (16,17) methods take advantage of Cas13 and Cas12a to detect limited sets of pathogen sequences with attomolar sensitivity in clinical samples. Our group recently demonstrated that recombinant Cas9 coupled with multiplexed sets of guide RNAs can be used for precision depletion of unwanted background sequences (18). We have now built on that work and developed FLASH (Finding Low Abundance Sequences by Hybridization).

**Figure 1.**
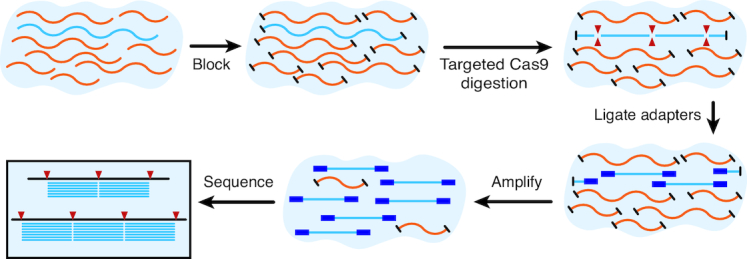
Overview of the FLASH method. Genomic DNA or cDNA is first blocked with phosphatase treatment and then digested with Cas9 complexed to a set of guide RNAs targeting genes of interest. Ligation of sequencing adaptors, amplification and sequencing follows.

This novel NGS targeted enrichment system has direct applicability to the challenge of AMR detection, among other applications. The FLASH technique uses a set of Cas9 guide RNAs designed to cleave sequences of interest into fragments appropriately sized for Illumina sequencing (Figure 1). Input genomic DNA or cDNA is first blocked by phosphatase treatment and then digested with Cas9 complexed to this set of guide RNAs. The resulting cleavage products are thus made competent for ligation of universal sequencing adapters. With the ensuing amplification, the targeted sequences are enriched over background and made ready for binding to the sequencing flow cell. This method goes beyond other CRISPR-based diagnostic tools in that it enables high levels of multiplexing (thousands of targets) and is reinforced by the precision and sequence identity confirmation that is inherent in a traditional NGS readout. We highlight two uses of FLASH-NGS in the realm of drug-resistant infections: the burden of antimicrobial resistance genes in pneumonia-causing gram-positive bacteria and drug resistance in the malaria parasite *Plasmodium falciparum*.

## MATERIALS AND METHODS

To choose optimal guide RNA targets for FLASH, we developed a flexible computational tool called FLASHit. Given a set of target genes, this tool first defines targetable 20-mer Cas9 sites, applying certain exclusion criteria (see Supplementary Methods). It then takes advantage of homology between genes to design a relatively small set of guide RNAs that provides a relatively high sequence coverage (Supplementary Figure S1). While a single FLASH-derived fragment is sometimes sufficient to uniquely identify an AMR gene, we designed guides to cut each gene into multiple fragments, both to increase the probability of detection in the case of a single nonfunctional guide RNA or unanticipated SNP and to maximize the ability to detect both known and unknown sequence variants. This was achieved by solving a mixed integer program with the objective of maximizing the number of inter-guide inserts of optimal Illumina NGS length (200–300 bp) covering each gene while minimizing the number of guide RNAs (see Supplementary Methods and github documentation). FLASHit is freely available at github.com/czbiohub/flash.

### Bacterial AMR FLASH

In order to construct a limited pilot set of guide RNAs that would be compatible with a more comprehensive future set, FLASHit was first used to design a set targeting the full collection of 3624 clinically relevant AMR-related genes derived from the CARD (19) and ResFinder (20) databases, merging exact duplicates (Supplementary Table S1). This set contained 5513 guide RNAs (Supplementary Table S2). For pilot experiments, a subset of these sequences was chosen to target 127 clinically relevant AMR genes present in *Staphylococcus aureus* and other gram-positive bacteria (Supplementary Table S3), including 118 acquired resistance genes and 9 chromosomal genes capable of carrying drug resistance-conferring point mutations (indicated in Supplementary Table S4). The latter mainly represent highly conserved genes and serve two functions: to determine the presence of a given bacterial species (even if no acquired resistance genes are present) and to identify resistance point mutations. This set contained 532 target sequences (indicated as ‘staph’ in Supplementary Table S2), with the majority of genes containing at least four target sites (Supplementary Figure S1). DNA templates for producing crRNAs (CRISPR RNAs) for each target were synthesized, transcribed separately, then purified and pooled.

For the cultured isolate experiments, DNA was isolated from six clinical *S. aureus* isolates and sequenced in triplicate with traditional NGS (NEBNext Ultra II FS DNA-Seq kit) and FLASH-NGS using the pilot guide RNA set described above, to a sequencing depth greater than 0.5M reads for each replicate. For a detailed FLASH-NGS protocol, see Supplementary Methods. Briefly, 5' phosphate groups were enzymatically cleaved using rAPid alkaline phosphatase which was subsequently deactivated with sodium orthovanadate. The dephosphorylated DNA was added to a master mix containing the CRISPR/Cas9 ribonucleoprotein complex and incubated at 37°C for 2 h. The Cas9 was deactivated with proteinase K and removed with a SPRI bead purification Samples were dA-tailed and adapter-ligated using the NEBNext Ultra II reagents and protocols. Following two SPRI bead purifications to remove adapter dimer, samples were indexed with 22 cycles of PCR using dual unique TruSeq i5/i7 barcode primers. Individual libraries were pooled and size selected for fragments between 250–600; pools that were too low in concentration were subsequently amplified using up to 5 cycles of PCR. All libraries were sequenced on Illumina MiSeq or NextSeq instruments. Data were demultiplexed, quality filtered with PriceSeqFilter (21), aligned to the 127 targeted *S. aureus* genes using Bowtie 2 (22), and tallied up with custom python scripts. Unless otherwise noted, all reported data represents reads per million (rpM) averaged across triplicates. Filtering and alignment results, including no-template controls, are provided in Supplementary Table S5.

In the direct clinical sample experiments, for each sample 25 ng of DNA or cDNA was subjected to NGS and FLASH-NGS following the protocol outlined above and described in detail in Supplementary Methods. Data were aligned to our pilot set of 127 targeted gram-positive genes using Bowtie 2 (22). Filtering and alignment results, including no-template controls, are provided in Supplementary Table S5.

### Plasmodium FLASH

Six *P. falciparum* genomic loci with drug-resistance associations (D-01 to D-06), 25 with high population diversity (P-01 to P-25) and 17 microsatellite sites (M-01 to M-17) were selected for FLASH-NGS (Supplementary Figure S6). A single guide RNA target site was chosen from each side of each locus such that PE150 sequencing of the resulting insert would yield coverage of all SNPs of interest (Supplementary Table S8). In order to best simulate clinically relevant samples, dried blood spots (DBSs) representing three different mixtures of the culture adapted *P. falciparum* strains U659, HB3 and D10 were prepared by spotting 20 μl of blood containing 10 000 parasites/μl onto filter paper. DNA was subsequently extracted and amplified by selective whole genome amplification (sWGA) using custom primers (23). One hundred nanograms of DNA from each sample was subjected to FLASH-NGS in the manner described above, using the *P. falciparum* guide RNA set. This was repeated for three independent sWGA reactions from each of the three different mixtures, as well as each of the three strains alone. We also sequenced sWGA-amplified mixed-strain blood spots without FLASH as a control. Each dataset consisted of at least 2M PE150 reads. Reads were aligned to the Pf3D7 genome (PlasmoDB version 28 (24)) using Bowtie 2 (22) and further analysis was done using SAMtools (25), BEDTools (26) and custom python and R scripts. Filtering and alignment results, including no-template controls, are provided in Supplementary Table S5.

## RESULTS AND DISCUSSION

### Assessing FLASH using cultured bacterial isolates

**Figure 2.**
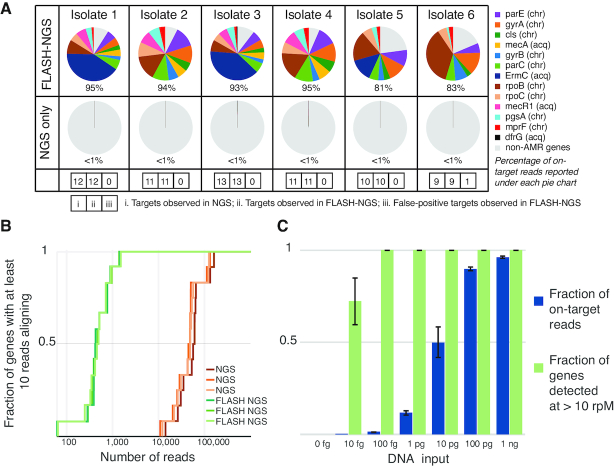
Results of FLASH on cultured isolates. (**A**) Proportion of targeted genes detected in FLASH-NGS and NGS libraries of six *S. aureus* isolates. Average of three replicates. chr: chromosomal gene; acq: acquired gene. Numbers in boxes represent, in order for each isolate: i. number of targets present (based on NGS); ii. number of expected targets observed in FLASH-NGS; iii. number of false-positive targets observed in FLASH-NGS (false positive not depicted in pie chart). (**B**) For isolate 1 with FLASH, <2000 sequencing reads were needed to achieve coverage of each targeted gene by at least 10 reads. Over 100 000 reads were needed to achieve the same coverage with NGS alone. (**C**) The fraction of targeted reads relative to background decreases substantially below 100 pg of DNA input; however, with as little as 100 fg input (∼35 copies of the *S. aureus* genome), the full set of targeted genes was detected at 10 rpM or greater. Bars and error bars represent mean and standard deviation of three replicates.

FLASH performance for AMR gene sequence identification was first evaluated in the context of cultured bacterial isolates. DNA from six clinical *S. aureus* isolates was sequenced in triplicate with traditional NGS and FLASH-NGS using the pilot guide RNA set described above. As expected, all nine chromosomal genes were recovered in all six isolates by NGS. Each isolate also contained between zero and four acquired resistance genes. For all isolates, every gene identified by NGS was also identified by FLASH-NGS above a threshold of 1000 rpM (Figure 2A). In a single case, one false positive gene was identified above this threshold with FLASH-NGS: ErmC at 6461 rpM in isolate 6. We believe this to be the result of cross-contamination from another isolate, either during library prep or on the sequencer, as ErmC was one of the two most abundant genes identified across all FLASH-NGS isolates (Figure 2A and Supplementary Table S5). This result highlights the need for caution when multiplexing samples using extremely sensitive amplification techniques such as FLASH. To summarize, in this experiment on cultured isolate DNA (where the ground truth was given by unbiased NGS), we observed zero false negatives and one false positive when considering 127 target genes across six samples.

The FLASH-NGS results were consistent with phenotypic testing, with the exception of one ciprofloxacin resistant isolate, and three instances of non-*ermC* clindamycin resistance – these exceptions were to be expected given that our pilot target set did not cover the full spectrum of ciprofloxacin or clindamycin resistance elements (Supplementary Table S6). On average, 90.1% of reads mapped to target genes in FLASH samples, compared to 0.3% mapping to these genes with NGS alone. This represented a 293-fold increase in average rpM of targeted genes (Figure 2A). For each FLASH-NGS sample, a sequencing depth between 500 and 5000 reads was sufficient to recover 10 or more reads per gene for 100% of targeted genes. For NGS alone, at least 100-fold higher sequencing depth was required to achieve this minimal threshold (Figure 2B and Supplementary Figure S2).

Several parameters may affect FLASH-NGS performance, including the complexity of the guide RNA set, the amount of input DNA, and the amount of Cas9 protein. The effect of guide RNA pool complexity was tested using an extended set of 2226 guides (transcribed as a single pool) (Supplementary Figure S3, Supplementary Table S2). On-target performance remained comparable, with an observed 90.6% recovery rate. To determine the limits of input nucleic acid for FLASH profiling, the mass of DNA from isolate 1 was progressively lowered to as little as 10 femtogram (Figure 2C). The fraction of FLASH-derived reads dropped below 50% at 10 pg (replaced mostly by reads corresponding to *Escherichia coli* derived from the Cas9 preparation itself; see Supplementary Table S5). Despite fewer on-target reads, all targeted genes were still covered by at least 10 rpM at 100 fg (∼35 *S. aureus* genome copies in 30 μl, or 1.9 aM), and over half of them were covered at 10 fg (0.19 aM). The amount of input Cas9 protein had little effect on the enrichment of target sequences down to 0.4pmol, which corresponds to ∼50 copies of each Cas9-guide RNA complex per *S. aureus* genome copy. This represents a materials cost of < $1 US when using commercially available Cas9 and guide RNAs transcribed from crRNA templates in a pool (Supplementary Figure S4).

We examined the *S. aureus* isolate data to understand why a small number of guide RNAs yielded few or no reads (Supplementary Table S7). Whole genome sequence data was used to determine the sequences at the target locations for all *S. aureus* isolates. Considering every target site in every gene present in each of the six isolates, there were a total of 622 target sequences present in this set of experiments (approximately 100 for each isolate). A total of 94.4% (587 of 622) target sites were readily detectable in FLASH-NGS experiments, using an arbitrary but conservative detection threshold of 1 rpM across the three replicates. Of the remaining 35 target sites, we noted that nine (25.7%) harbored mutations within the targeting sequence. This reinforces the need to build in additional redundancy by selecting multiple target sites per gene, a key feature of the FLASHit program. It is unclear exactly why the remaining 26 guide RNAs failed. While numerous tools exist to assess the efficiency of different guide RNA sequences (27,28), they are mostly concerned with *in vivo* genome editing capabilities, rather than *in vitro* cutting activity. We expect that as more FLASH data is collected, *in vitro* guide failure patterns will emerge.

### FLASH enrichment directly from clinical samples

**Figure 3.**
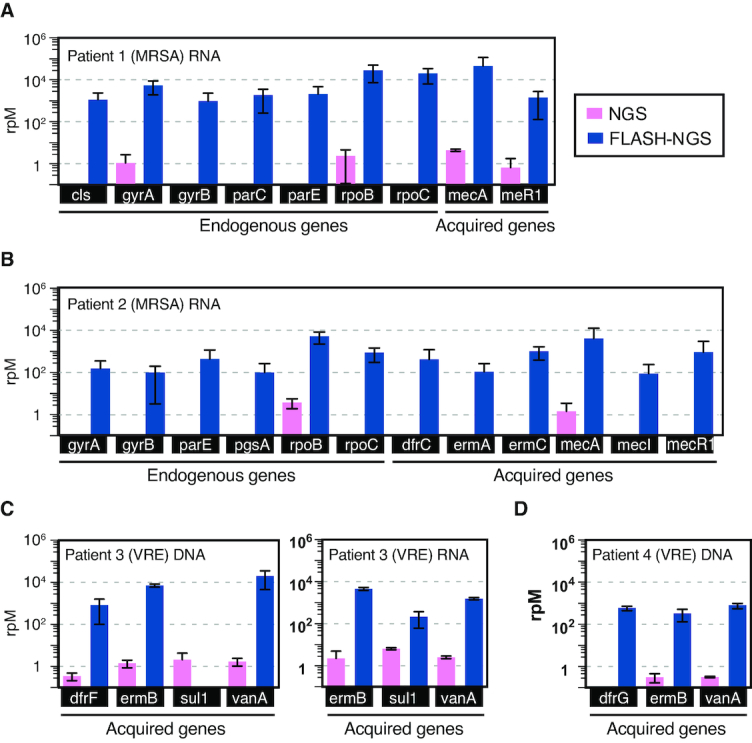
Results of FLASH on respiratory samples. Number of reads aligning to targeted genes in NGS and FLASH-NGS sequencing experiments on respiratory fluid samples from (**A**) patient 1, (**B**) patient 2, (**C**) patient 3 and (**D**) patient 4. Average of three or six replicates. Bars and error bars represent mean and standard deviation of three to six replicates (see Supplementary Table S5).

Culture-based infectious disease diagnostics often fail to provide actionable data due to challenges with growing fastidious and slowly replicating microbes such as mycobacterial and fungal pathogens, and administration of antibiotics prior to sampling. We thus sought to assess the performance of FLASH-NGS for detecting AMR gene targets directly from four patient samples with culture-confirmed drug resistant infections (Supplementary Table S6), including from both DNA and RNA preparations. Each was subjected to NGS and FLASH-NGS following the protocol outlined above and described in detail in Supplementary Methods. Data were aligned to our pilot set of 127 targeted Gram-positive genes using Bowtie 2 (22). Filtering and alignment results, including no-template controls, are provided in Supplementary Table S5. Figure 3 shows reads per million for each targeted gene in each clinical sample detected with NGS alone and FLASH-NGS. All data represent averages of three to six experiments. In the context of patient samples, where the microbial component is a minority of the nucleic acid, the proportion of on-target sequences was lower than for cultured isolates (Supplementary Figure S5). However, the mean enrichment over NGS was >5000-fold (range 563-fold to 13 244-fold per sample), and in many cases FLASH-NGS detected genes that were unobservable with NGS alone, as described below. Due to the difficulty of assessing true positivity in the context of these clinical samples, it is difficult to determine sensitivity and specificity of the FLASH method in this context. We note that studies currently underway on broader sets of clinical samples will address this question more directly.

Patient 1 (Figure 3A) was hospitalized for culture-positive methicillin-resistant *S. aureus* (MRSA) pneumonia. FLASH-NGS of RNA extracted from mini-bronchial alveolar lavage (mBAL) identified the *mecA* gene, which explains the methicillin resistance observed in the *S. aureus* isolated from this patient (Supplementary Table S6), at over 20 000 rpM. Seven *S. aureus* chromosomal genes were also detected (*cls, gyrA, gyrB, parC, parE, rpoB* and *rpoC*). Patient 2 was admitted with fatal pneumonia that was culture positive for MRSA, *Citrobacter* and *Pseudomonas* (Figure 3B). FLASH-NGS performed on RNA extracted from tracheal aspirate (TA) detected six *S. aureus* chromosomal genes (*gyrA, gyrB, parE, pgsA, rpoB* and *rpoC*) plus acquired genes conferring resistance to trimethoprim-sulfamethoxazole (TMP-SMZ) (*dfrC*), macrolides (*ermA* and *ermC*) and methicillin (*mecA* and its regulators *mecI* and *mecR1*). Given the polymicrobial nature of this patient's infection, it was not possible to say with certainty whether the acquired resistance genes originated with the phenotypically multidrug resistant *S. aureus* (Supplementary Table S6) or from another species.

Patient 3 was admitted for a lower respiratory tract infection. Vancomycin-resistant *Enterococcus faecium* (VRE) was identified by culture. FLASH-NGS of DNA from mBAL fluid identified the *vanA* gene, which confers vancomycin resistance. Resistance to macrolides and TMP-SMZ are widespread in *Enterococci* and thus identification of *ermB* (macrolide resistance), and of *dfrF* (TMP-SMZ resistance) was not surprising. With FLASH-NGS of RNA from the same sample, *dfrF* was not detected (Figure 3C), likely indicating this gene was not being expressed. We note that *sul1* was detected in patient 3 DNA by NGS but not FLASH-NGS. Examination of all read pairs aligning to this gene in this patient revealed that they exclusively mapped to a 250 bp region containing only a single FLASH target site. This does not preclude FLASH enrichment: in RNA, thousands of read pairs were derived from this site on one side and nonspecific cleavage on the other side. Why this was not observed in the DNA samples is unknown. Overall, the patient 3 results demonstrate the utility of testing both nucleic acid types. RNA profiling provides information on what genes are active, and relatively high RNA expression levels may enhance the probability of detection. DNA profiling may identify relevant genotypes even in the absence of expression, results that will at times be clinically relevant given the inducible nature of some AMR mechanisms

Patient 4 (Figure 3D) was admitted for VRE bacteremia and also found to have vancomycin-resistant *E. faecium*; FLASH-NGS of DNA from this patient's TA identified *vanA* (vancomycin resistance) as well as *ermB* (macrolide resistance) and *dfrG* (TMP-SMZ resistance).

In addition to acquired resistance elements, antimicrobial resistance can also be conferred by single point mutations in chromosomal genes. Notably, FLASH-NGS can recover SNP data simultaneously with presence/absence data for acquired resistance genes located on mobile genetic elements. For example, RNA from patient 2 shows wildtype sequence for all but one rifampicin resistance SNP location in the *rpoB* gene. At position 481, where wildtype was histidine, we found a mixture of 33% wildtype and 77% H481Y mutation out of a total of over 20 000 reads. H841Y *rpoB* has been described as rifampicin resistant (29). We also found 97.3% of the over 500 reads mapping to *gyrA* position 84 in this patient represented the S84L mutation, which explains the ciprofloxacin resistance observed in the *S. aureus* isolated from this patient (Supplementary Table S6) (19). The genetic mixtures may indicate a coinfection with more than one strain of *S. aureus* in this patient, or evolving resistance mutations.

### FLASH-NGS for *Plasmodium falciparum* drug resistance and strain diversity

**Figure 4.**
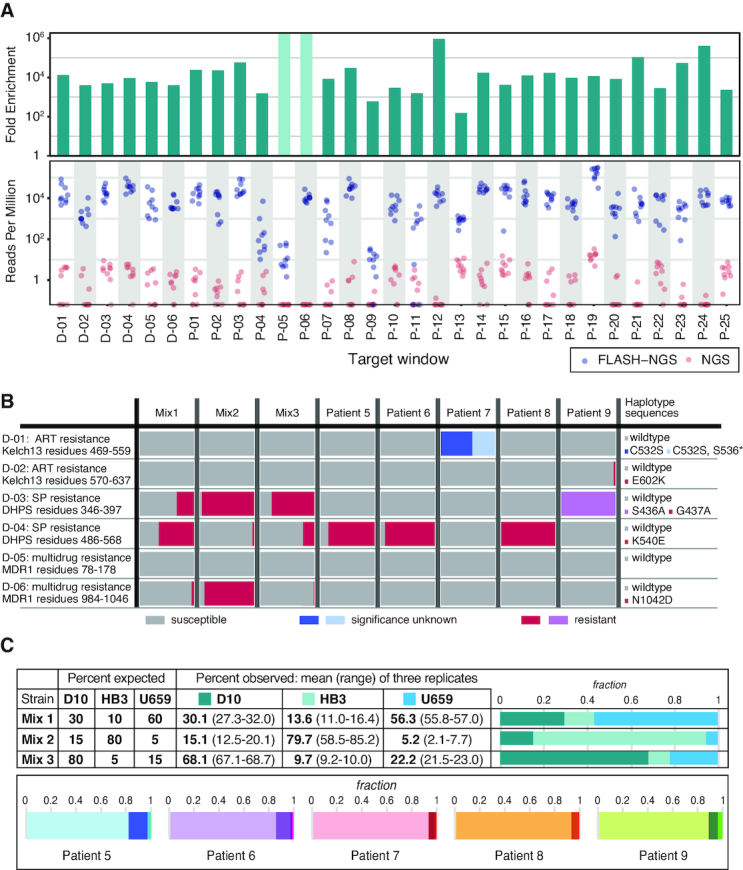
Results of FLASH on dried blood spots. (**A**) Dried blood spots (DBSs) from malaria lab strains were sequenced using either NGS or FLASH-NGS. Reads per million is plotted for each window for nine FLASH-NGS samples (three strain mixtures, each in triplicate, blue) and nine equivalent NGS samples (pink). Top panel indicates fold enrichment (average FLASH-NGS rpM divided by average NGS rpM). Light green bars in the upper panel represent windows for which no haplotype-determining read pairs were found. (**B**) Both lab strain mixtures and patient samples were evaluated for the presence of drug resistant haplotypes. Bars indicate mean of three replicates. (**C**) Target windows with sequences that distinguish the lab strains D10, HB3 and U659 were used to estimate strain ratios in the three mixtures. For the clinical DBS samples, the number of sequencing reads attributable to different haplotypes at each window was determined using SeekDeep. The average proportion of haplotypes at each of the maximum-haplotype containing windows is depicted. Bars indicate mean of three replicates.

The challenge of multiplex drug resistance detection is not limited to prokaryotes. We adapted FLASH to identify malaria strain variants in the context of mixed infections, an important challenge for malaria control and elimination efforts and a modality that could be extended to many areas of epidemiology. Six *P. falciparum* genomic loci with drug-resistance associations (D-01 to D-06), 25 with high population diversity (P-01 to P-25) and 17 microsatellite sites (M-01 to M-17) were selected for FLASH-NGS (Supplementary Figure S6) and a single guide RNA target site was chosen from each side of each locus as described in Methods. DBSs representing three different mixtures of the culture-adapted *P. falciparum* strains U659, HB3 and D10 were prepared and then sequenced with NGS and FLASH-NGS as described in Methods. On average we observed 85.6% on-target reads with FLASH-NGS on these samples, compared with <0.02% on-target without FLASH (Supplementary Figure S7). For the triple strain samples, the 31 D and P windows targeted were enriched from two to more than five orders of magnitude over traditional NGS when averaging all experiments from all strain mixtures (*n* = 9, three replicates each of three different strain mixes) and considering only read pairs in which all haplotype-defining SNPs in a particular window were sequenced (Figure 4A).

We also applied FLASH-NGS with the same guide RNA set to DBSs from patients in the Zambezi region of Namibia (patients 5–9). DBSs were selected for having a parasite density >10 000/μl (determined by qPCR, see Supplementary Methods). These were extracted, amplified with sWGA and subjected to FLASH-NGS in triplicate. The six targeted drug resistance windows represented loci in the genes *kelch-13, dhps* and *mdr1* (Figure 4B). Using FLASH-NGS, at least 2500 sequencing reads were obtained for each window for each sample (it should be noted, however, that in this pilot guide RNA set only two windows were targeted for each gene, so additional non-targeted mutations could have been missed). Mutations in *kelch-13* confer resistance to artemisinin. No *kelch-13* mutations were detected in the lab strains. Patient 7 had a *kelch-13* C532S mutation at 100%; this has been observed before but the significance is unknown (30). In addition, 41.4% of reads in this patient had a stop codon mutation at position 536. Patient 9 had an E602K mutation present in 2.8% of reads, suggesting a low frequency of artemisinin resistance. Mutations in the *dhps* gene conferring resistance to the antimalarial Sulfadoxine/Pyrimethamine (specifically the resistance-conferring K540E mutation) are relevant for public health in Africa as their prevalence is used by the WHO to determine intermittent presumptive therapy (IPT) regimens. We detected the resistant G437A haplotype at variable levels (along with the susceptible wildtype haplotype) in all three lab strain mixtures. Four of the patients had mutations in this region as well: patients 5, 6 and 8 had the resistant K540E mutation at 85–100%, and patient 9 had the resistant S436A mutation at 100%. This result was expected in this population (31). Finally, we targeted the multidrug resistance locus *mdr1*. The lab strain mixtures showed variable levels of the N1042D resistance mutation, but only wildtype *mdr1* sequences were detected in the patient samples.

*P. falciparum* haplotypes derived from FLASH-NGS experiments on lab strain mixtures were analyzed to determine whether minor strains were detectable and whether strain ratios could be accurately recovered. Twenty-one of the 48 windows uniquely distinguished all three strains from each other (Supplementary Figure S8). In all three mixtures, we reliably detected all three strains. To estimate the haplotype ratios from these data, we averaged the percentages across these windows (Figure 4C). Supplementary Figure S8 depicts the percentages of each strain represented in each window (dots) along with the median and interquartile range across all windows (boxes). While any individual window was an imprecise estimate of haplotype ratio, averaging across the 21 windows converged on an accurate estimate for each of the three replicates of each of the three strain mixtures.

For the clinical samples, the number of variable haplotypes was determined for each window using SeekDeep (32), and is depicted in Figure 4C and Supplementary Figure S9. Patients 5 and 6 have two windows each for which four unique haplotypes were identified, and patients 7, 8 and 9 have at least 4 windows each for which three unique haplotypes were identified. Averaging strain percentages across only these maximum haplotype windows indicated that the primary haplotype comprised between 82% and 94% of parasites, with the 2–3 additional haplotypes making up the remainder (Figure 4C). Notably, none of these patients shared a complete set of identical primary haplotype sequences, suggesting that five different strains accounted for the primary infections in these five individuals (although some windows did share identical sequences) (Supplementary Figure S9).

## CONCLUSION

In conclusion, we have developed a targeted sequencing method that is fast, inexpensive, has high multiplexing capacity, and is nimble enough to target virtually any sequence of interest without optimization. It is the efficiency, specificity and programmability of the CRISPR/Cas9 system that allows this functionality. Highly multiplexed detection of antimicrobial resistance genes in patient samples is an important use case for FLASH-NGS; however, it is by no means the only application area for this technique. Detection of mutations in cancer, rare mosaic allele detection, targeted transcriptomics from clinical samples, enrichment of microbiome components from complex mixtures, and recovery of targeted transcripts from single cell sequencing libraries are among other possible applications that may be explored in future work.

## DATA AVAILABILITY

Microbial sequence data are available on SRA in BioProject ID PRJNA493248. Code is available on github.com/czbiohub/flash.

## Supplementary Material

gkz418_Supplemental_Files

## Notes

Present address: Jenai Quan, Northwestern University Feinberg School of Medicine, Chicago, IL 60611, USA.
