# Supplementary material for: FLASH: a next-generation CRISPR diagnostic for multiplexed detection of antimicrobial resistance sequences
Source: Nucleic Acids Res. 2019 May 22;47(14):e83. doi: 10.1093/nar/gkz418 (PMC6698650; doi:10.1093/nar/gkz418)
Supplement: gkz418_Supplemental_Files [file gkz418_supplemental_files.zip › FLASH_supplementary_NAR_R2.docx]

**FLASH: A next-generation CRISPR diagnostic for multiplexed detection of antimicrobial resistance sequences**

Jenai Quan^1,2^, Charles Langelier^3^, Alison Kuchta^4^, Joshua Batson^1^, Noam Teyssier^3^, Amy Lyden^1^, Saharai Caldera^3^, Aaron McGeever^1^, Boris Dimitrov^5^, Ryan King^5^, Jordan Wilheim^6^, Maxwell Murphy^6^, Lara Pesce Ares^1^, Katherine A. Travisano^1^, Rene Sit^1^, Roberto Amato^7^, Davis R. Mumbengegwi^8^, Jennifer L. Smith^9^, Adam Bennett^9^, Roly Gosling^9^, Peter M. Mourani^10^, Carolyn S. Calfee^11^, Norma F. Neff^1^, Eric D. Chow^2,12^, Peter S. Kim^1,13,14^, Bryan Greenhouse^1,6^, Joseph L. DeRisi^1,2^, Emily D. Crawford*^1,2,15^

**Materials and Methods**

Generation of DNA from *Staphylococcus aureus* isolates:

Bacterial cultures were inoculated following standard clinical protocols for tracheal aspirate. Phenotypic antibiotic resistance was obtained using the Vitek2 system (Biomerieux, Marcy-l'Étoile, France) (Table S6). DNA from cultured isolates was extracted using either the Zymo Quick DNA extraction kit (Zymo Research, Irvine, CA) or the Qiagen AllPrep RNA/DNA kit (Qiagen, Hilden, Germany) according to manufacturer’s instructions.

Generation of DNA and cDNA from direct clinical respiratory specimens:

Excess tracheal aspirate (patients 1 and 4) or mini-bronchial alveolar lavage (patient 3) specimens were collected from patients enrolled in a study examining acute respiratory disease in critically ill adults according to University of California San Francisco Institutional Review Board protocol 10-02701. Tracheal aspirate specimen from patient 2 was collected from a patient enrolled in a study investigating factors predisposing to ventilator associated pneumonia in critically ill children according to University of Colorado Institutional Review Board protocol 14-1530.

DNA and RNA was extracted using the Qiagen AllPrep RNA/DNA kit on the QIAcube as described in (1). cDNA was generated from extracted RNA using Nugen Ovation v2 SPIA amplification kit (NuGen, San Carlos, CA, USA). Phenotypic antibiotic resistance was obtained using the Vitek2 system (Table S6).

*Plasmodium falciparum* dried blood spot extractions and selective whole genome amplification:

Individual asexual *P. falciparum* lab strains D10, HB3 and U659 were grown in human donor red blood cells (RBCs) at 2% hematocrit (percentage of isolated and cleaned RBCs in total culture volume) in RPMI 1640 media with 2mM L-Glutamine, 25 mM HEPES, 2 g/L sodium bicarbonate, 5 g/L AlbuMAX II Lipid-Rich BSA (Life Technologies, Carlsbad, CA, USA), 0.1 mM hypoxanthine, 50 mg/L gentamicin. Cultures were grown at 37°C, 5% O_2_, and 5% CO_2_. RBCs were collected from human donors under UCSF IRB number 10-3852. Parasitemia (percentage of parasite-infected RBCs out of total RBCs) and synchronization of the parasite culture was routinely monitored.

To simulate clinical dried blood spot (DBS) samples, cultured parasites were spotted onto Whatman filter paper with a parasite density of 10,000 parasites/spot as determined by % parasitemia and % hematocrit. This was done for each of the three lab strains (D10, HB3 and U659) individually, and also for cultures consisting of the three strains mixed at three different known percentages (Fig. 4C). Thus, a total of 6 DBS sample types were used, each in triplicate. Genomic DNA was isolated from the dried blood spots using the QiaAMP blood DNA mini kit from Qiagen.

Five samples were collected from symptomatic malaria cases in 2016 from the Zambezi region of Namibia as described (Tessema et al., in preparation). Briefly, samples were spotted on 3MM Whatman filter paper, dried and stored at -20°C until processing. Six mm hole punches from dried blood spots were extracted using the saponin Chelex method (2) and parasite density was quantified using varATS ultra-sensitive qPCR (3). Hole punching and extraction was done in triplicate for each of the five DBSs.

All DBS-extracted DNA samples underwent selective whole genome amplification (sWGA), an isothermal amplification technique that allows for preferential enrichment of the malaria genome over the human genome, as described previously with some modifications (4, 5). Briefly, a 50 µL sWGA PCR reaction consisting of a final concentration of 1X NEB phi29 reaction buffer, 1X Bovine Serum Albumin, 2.5 µM primer set 6A (4), 2.5 µM of primer set 10A (5), 2 mM dNTP mix with 70% AT and 30% GC ratio, 30 units of NEB phi29 DNA Polymerase, plus 20 µL of DNA directly from the Qiagen or Chelex extraction. Cycling conditions are as follows: 35°C x 5 min, 34°C x 10 min, 33°C x 15 min, 32°C x 20 min, 31°C x 30 min, 30°C x 16 hours, 65°C x 15 min, 6°C hold. Reactions were quantified by high sensitivity DNA Qubit (Thermo Fisher Scientific, Waltham, MA, USA).

Preparation of CRISPR/Cas9:

The CRISPR/Cas9 (Clustered Regularly Interspersed Short Palindromic Repeats/CRISPR associated protein 9) protein was generated as described in (6) From N to C terminus, the construct consisted of the following components: 6X HIS tag, maltose binding protein (MBP), S. pyogenes Cas9, 2X SV40 nuclear localization site (NLS), mCherry, 1X SV40 NLS.

Briefly, the Cas9 vector was expressed in BL21-CodonPlus (DE3)-RIL competent cells (Agilent, Santa Clara, CA, USA) for three hours at 16°C, after which cultures were centrifuged and frozen. Thawed cell pellets were later resuspended in lysis buffer (50 mM sodium phosphate pH 6.5, 350 mM NaCl, 10% glycerol, 1 mM TCEP) supplemented with protease inhibitors and microfluidized. The soluble fraction was purified on a heparin column on the GE AKTA Pure system, then concentrated down and further fractionated by size exclusion chromatography. The resulting pooled fractions were concentrated and stored at -80°C in 50% glycerol.

Guide RNA design, antimicrobial resistance genes:

We began with all antimicrobial resistance genes listed in the CARD and ResFinder databases, removing exact duplicates. Using FLASHit, we determined the set of all possible guide RNAs (100,931) with the following exclusion criteria: (a) no homopolymers of length greater than 5; (b) no runs of 2-base repeats greater than 3; (c) no internal hairpins; and (d) GC content between 25% and 75%. Reasoning that a guide that can bind a human sequence could be sequestered away from its pathogen target by the highly abundant human DNA in a metagenomic sample, FLASHit removes from consideration any guide that matches a human sequence with zero mismatches in the five PAM proximal bases (the seed region (7, 8)), with one or fewer mismatches in the 10 most PAM proximal bases, and with 2 or fewer mismatches in the full 20-mer. FLASHit also removes guides that match a sequence in the common *E. coli* expression strain BL21, used to produce the Cas9, to avoid false positives resulting from residual DNA bound to the enzyme. FLASHit was then used to define an optimized set of 5,513 guides from this set. The optimization method uses a mixed integer program (9) and is described in the github documentation. Characteristics of the resulting guide RNA set are depicted in Fig. S1.

Guide RNA design, *P. falciparum* drug resistance windows, population diversity windows, and microsatellite windows:

Areas of significant diversity across the malaria genome were identified by using custom R scripts to search for 250 bp regions with multiple single nucleotide polymorphisms (SNPs) having global minor allele frequencies of > 0.1. These targets have epidemiological diversity between multiple populations. Twenty-five of them were selected and are referred to as population diversity windows (P). Seventeen microsatellites regions (M) were also included. Six windows located at three key malaria drug resistance genes of interest (*kelch-13*, *dhps* and *mdr1*) were also selected, and are referred to as drug resistance windows (D). Potential target RNA binding sites were identified for each window. Pairs of guide RNAs surrounding each insert were chosen by hand based on coverage with PE150 sequencing (i.e. window size less than 300bp) and absence of knowns SNPs at the guide RNA target site. All guide RNA target sites had GC content between 5% and 50% and none contained a homopolymer greater than 9 bases in length or a series of greater than 5 dinucleotide repeats or greater than 3 trinucleotide repeats. Insert sizes ranged from 156 bp to 338 bp, with an average of 245 bp. Key SNP positions within each window were identified from MalariaGEN with a minor allele frequency (MAF) >= 0.001 in Africa (https://www.malariagen.net/projects/p-falciparum-community-project). Chromosomal locations of target windows are indicated in Fig. S6. Windows, guide RNA targets, and SNPs are indicated in Table S8.

Guide RNA preparation:

DNA oligonucleotide templates for CRISPR RNA (crRNA) and trans-activating CRISPR RNA (tracrRNA) were purchased from Integrated DNA Technologies (IDT, Coralville, IA, USA). crRNA template sequences were as follows (T7 RNA polymerase site is underlined):

5`TAATACGACTCACTATAGNNNNNNNNNNNNNNNNNNNNGTTTTAGAGCTATGCTGTTTTG3`

where the 20 Ns represent the 20 nucleotide target region. tracrRNA template sequence was as follows:

5`TAATACGACTCACTATAGGACAGCATAGCAAGTTAAAATAAGGCTAGTCCGTTATCAACTTGAAAAAGTGGCACCGAGTCGGTGCTTTTT3`

crRNAs were transcribed individually from their DNA template using T7 RNA polymerase for 2 hours at 37°C. Each reaction contained the following components: 100 ng crRNA DNA template or 8ug tracrRNA DNA template, T7 buffer (final concentrations 40 mM Tris pH 8.0, 20 mM MgCl2, 5 mM DTT, and 2 mM spermidine), NTPs (1mM each ATP, CTP, UTP, GTP), and 10 ng/µL T7 enzyme. Guide RNAs were purified using SPRI (Solid Phase Reversible Immobilization) magnetic beads. Individual crRNAs were pooled and stored as 80 µM single-use aliquots at -80°C. Immediately prior to use, pooled crRNAs were annealed with tracrRNA at an equimolar ratio to form 40 µM dual-guide RNA.

For the expanded set of 2,226 antimicrobial resistance (AMR) guide RNAs (Fig. S3), all guides were transcribed in a single pool.

Preparation of standard Next Generation Sequencing (NGS) Libraries:

RNA libraries from patient respiratory fluid: Five µL of RNA from each sample was reverse transcribed with the NuGEN Ovation v2 SPIA kit. The concentration of cDNA was quantified via high sensitivity DNA Qubit. End repair of 25 ng of cDNA was done as described in the NEBNext Ultra II protocol (New England Biolabs, Ipswitch, MA, USA).

DNA libraries from patient respiratory fluid: After extraction, DNA was quantified via high sensitivity DNA Qubit. Twenty-five nanograms of DNA was fragmented for 12.5 minutes at 37°C and end-repaired at 65°C using the NEBNext Ultra II FS DNA protocol.

DNA libraries from bacterial cultured isolates: Samples of between 10 fg and 25 ng of total DNA were fragmented for 5 minutes at 37°C and end-repaired at 65°C using the NEBNext Ultra II FS DNA protocol.

DNA libraries from sWGA of malaria DBS: One hundred nanograms of sWGA-amplified DNA was end-repaired as described in the NEBNext Ultra II protocol.

All libraries: Following end-repair, adaptor ligation at a 1:100 adaptor dilution was performed as described in the NEBNext Ultra II protocol. A SPRI bead purification was done at a sample:bead volume ratio of 1:1 and samples were eluted in 15 µL. The purified ligated products were indexed with 9-18 cycles of PCR using NEB Q5 polymerase and dual unique TruSeq i5/i7 barcode primers. A SPRI bead purification was done at a sample:bead volume ratio of 1:1. Individual libraries were analyzed using the High Sensitivity DNA Bioanalyzer kit and pooled based on library shape and according to the concentration of DNA within the range of 250 to 600 base pairs. Pooled samples were size selected for fragments between 250-600 bp using the BluePippin 2% agarose gel cassette (Sage Science, Beverly, MA, USA). The BluePippin product was SPRI bead purified at a sample:bead volume ratio of 1:1.4 and eluted in 15 µL of water. Pooled library quality was assessed on the Bioanalzyer (Agilent technologies, Santa Clara, CA). Pools that were too low in concentration were amplified using up to 5 cycles of KAPA HiFi HotStart Real-time PCR (Kapa Biosystems, Roche, Basel, Switzerland) according to the manufacturer’s protocol and cycling conditions. Amplified samples were SPRI bead purified using a sample:bead volume ratio of 1:1.4 and eluted in 15 µL. Pooled libraries were quantified using High Sensitivity DNA Qubit. Final library quality was assessed using on the Bioanalyzer and additional SPRI bead purifications were performed if adapter dimer was present. Final libraries were quantified using qPCR.

Preparation of FLASH-NGS Libraries:

The 5` phosphate groups of 25 ng DNA or cDNA from cultured isolates or respiratory fluid, or 100 ng of sWGA from malaria DBSs, were enzymatically cleaved using rAPid alkaline phosphatase (Sigma Aldrich, St. Louis, MO, USA) for 30 mins at 37°C according to the manufacturer’s instructions. The phosphatase enzyme was deactivated with one unit of sodium orthovanadate. The dephosphorylated DNA was added to a master mix containing the CRISPR/Cas9 ribonucleoprotein complex. The final mixture was a 30 µL reaction of dephosphorylated DNA, Cas9 buffer (50 mM Tris pH 8.0, 100 mM NaCl, 10 mM MgCl_2_, and 1 mM TCEP), 500 nM Cas9, and 6 µM appropriate dual guide RNAs. The mixture was incubated at 37°C for 2 hours. The Cas9 was deactivated by adding 1 µL of proteinase K and incubating at 37°C for another 15 minutes. A SPRI bead purification at a sample:bead volume ratio of 1:1.7 was conducted and samples were eluted in 50 µL. Samples were dA-tailed using the NEBNext dA-Tailing Module. Adaptor ligation at a 1:100 adaptor dilution was performed as described in the NEBNext Ultra II protocol. Two SPRI bead purifications were done at a sample:bead volume ratio of 1:1 and samples were eluted in 15 µL. The purified ligated products were indexed with 22 cycles of PCR using NEB Q5 polymerase and dual unique TruSeq i5/i7 barcode primers. A SPRI bead purification was done at sample:bead volume ratio of 1:1. Individual libraries were analyzed using the High Sensitivity DNA Bioanalyzer kit and pooled based on library shape and according to the concentration of DNA within the range of 250 to 600 base pairs. Pooled samples were size selected for fragments between 250-600 bp using the BluePippin 2% agarose gel cassette. The BluePippin product was SPRI bead purified at a sample:bead volume ratio of 1:1.4 and eluted in 15 µL. Pooled library quality was assessed on the Bioanalzyer. Pools that were too low in concentration were amplified using up to 5 cycles of KAPA HiFi HotStart Real-time PCR according to the manufacturer’s protocol and cycling conditions. Amplified samples were SPRI bead purified using a sample:bead volume ratio of 1:1.4 and eluted in 15 µL. Pooled libraries were quantified using High Sensitivity DNA Qubit. Final library quality was assessed using on the Bioanalyzer and additional SPRI bead purifications were performed if adapter dimer was present. Final libraries were quantified using qPCR.

Sequencing:

All libraries were sequenced on Illumina MiSeq or NextSeq instruments (Illumina, San Diego, CA, USA). Raw sequence data for all isolate experiments is available on SRA. Sequence reads mapping to AMR targets or to the *P. falciparum* genome are also available on SRA. Raw read counts, filtered read counts, and number of reads aligning to targets for all experiments are available in Table S5.

Cultured S*. aureus* isolate data analysis:

Datasets were demultiplexed and then filtered with PriceSeqFilter (10) using the flags *-pair both rqf 85 0.98 -rnf 90*. This removes any read pairs for which either read contains less than 85% of nucleotides with a probability of being called correctly of at least 0.98, or for which either read has less than 90% of nucleotides called (more than 10% Ns). Filtered datasets were aligned to the 127 target genes using Bowtie 2 with the flags *-a -X 1000 --very-sensitive*. The *-a* flag forces all alignments to be considered and reported. The resulting .sam file was filtered using SAMtools (11) with *samtools view -F 256* to retain only the highest-scoring alignment for each read pair. Reads aligning to the 127 target genes were tabulated with custom python scripts. NGS data was used to identify which of the 127 genes was present in each of the six isolates at 10 rpM (reads per million) or greater, and only those genes were included in the analyses. The 127 target genes included two different *parE* sequences from two different strains of *S. aureus*; reads aligning to these two genes were summed to get a *parE* total. These results are presented in Figs. 2, S2, S3 and S4. The additional guide RNAs used in Fig. S3 are marked as “extended” in Table S2.

To determine *E. coli* contamination from the Cas9 prep, filtered datasets were aligned to the *E. coli BL21* genome using Bowtie 2 with the *-very-sensitive-local* flag.

NGS data was used to identify the sequence of each guide RNA target site present in each of the isolates. Only target sites with at least 1 rpM (averaged over the three replicates) having an overlap of at least one base with the site sequence in the NGS isolate data were included in this analysis. Mutations were identified and are indicated in Table S7. For each guide site in each isolate, the alignment files were examined and reads that aligned with a start position within 2 bp of the cut site were identified and tallied as FLASH-derived reads. These results are presented in Table S7.

Patient respiratory fluid data analysis:

Datasets were demultiplexed and then filtered with PriceSeqFilter using the flags *-pair both -rqf 85 0.98 -rnf 90*. Filtered datasets were aligned to the 127 target genes using Bowtie 2 with the flags *-a -X 1000 --very-sensitive*. The *-a* flag forces all alignments to be considered and reported. The resulting .sam file was filtered with *samtools view -F 256* to retain only the highest-scoring alignment for each read pair. Reads aligning to the 127 target genes were tabulated with custom python scripts. These results are presented in Fig. 3 and S5. Genes were plotted in Fig. 3 only if an average of 100 rpM in FLASH-NGS samples and/or 1 rpM in NGS samples aligned to them. The SNP analysis of the *rpoB* and *gyrA* genes from Patient 1 was done using the variant call feature in Geneious (12).

*P. falciparum* data analysis:

Enrichment of *P. falciparum* target windows: Datasets were demultiplexed and filtered with PriceSeqFilter (10) using the flags *-pair both -rqf 85 0.98 -rnf 90*. Fig. S7 was generated by using Bowtie 2 to align filtered datasets to Pf3D7 reference sequences of the 48 target windows.

Enrichment of haplotype-determining SNPs in *P. falciparum* target windows: Filtered datasets were also aligned to the Pf3D7 genome (PlasmoDB version 28 (13)) using Bowtie 2 with the flag *--very-sensitive-local*. The resulting .bam files were filtered to contain only properly paired reads with *samtools view* *-f 0x2,* and then converted to .bedpe files with *bedtools bamtobed -bedpe* (14)*.* These .bedpe files were reduced to .bed files describing the outer spans of each read pair using awk (*gawk '{ print $1 "\t" $2 "\t" $6 "\t" $7 "\t" $8 "\t" $9 "\t" $10 }'*). A separate .bed file was created containing chromosomal locations encompassing only the key SNP positions (identified by MalariaGEN has having an MAF >= 0.001 in Africa) within each of the 48 target windows (rows denoted “snp_range” in Table S8). Finally, *bedtools intersect* was used to count the number of read pair spans which covered 100% of a target window using the flags *-f 1 -c* for each sample. M (microsatellite) windows were omitted from this analysis. These results are presented in Fig. 4A and represent 9 NGS and 9 FLASH-NGS experiments: three replicates of each of three triple strain mixtures.

Determination of *P. falciparum* haplotype ratios: Single strain NGS datasets from each of the three lab strains was used to determine the sequence of each target window in each strain. Twenty-one of the 48 windows had unique sequences for each of the three strains (P-01, P-03, P-05, P-06, P-07, P-08, P-11, P-12, P-13, P-14, P-15, P-17, P-18, P-21, P-23, P-24, P-25, M-12, M-15, M-17, and M-04). A fasta file containing each sequence for each of these windows was created, and for all NGS and FLASH-NGS experiments, filtered datasets were aligned to this file using Bowtie 2 with the flags *-a -X 1000 --very-sensitive*. The *-a* flag forces all alignments to be considered and reported. The resulting .sam files were filtered with *samtools view -F 256* to retain only the highest-scoring alignment for each read pair. The number of reads aligning to each variant of each target window was tabulated using grep commands. These results are presented in Figs. 4C and S8.

Haplotype analysis of malaria patient samples: Datasets were demultiplexed and then filtered with PriceSeqFilter (10) using the flags *-pair both -rqf 85 0.98 -rnf 90*. Haplotype analysis was carried out using SeekDeep (15) with default parameters. These results are presented in Fig. 4B.

*P. falciparum* drug resistance mutations in lab samples and patient samples: To evaluate drug resistance SNPs, all filtered samples were aligned to the 6 drug resistance windows using Bowtie 2 with the *--very-sensitive-local* flag and analyzed with the variant call feature in Geneious. These results are presented in Fig. 4C.

Acknowledgements

This publication uses data from the MalariaGEN *P. falciparum* Community Project (www.malariagen.net/projects/p-falciparum-community-project). MalariaGEN’s genome sequencing was performed by the Wellcome Trust Sanger Institute and the Community Projects is coordinated by the MalariaGEN Resource Centre with funding from the Wellcome Trust (098051, 090770)

**REFERENCES**

1. Langelier,C., Kalantar,K.L., Moazed,F., Wilson,M.R., Crawford,E., Deiss,T., Belzer,A., Bolourchi,S., Caldera,S., Fung,M., *et al.* (2018) Integrating Host Response and Unbiased Microbe Detection for Lower Respiratory Tract Infection Diagnosis in Critically Ill Adults. 10.1101/341149.

2. Plowe,C.V., Djimde,A., Bouare,M., Doumbo,O. and Wellems,T.E. (1995) Pyrimethamine and Proguanil Resistance-Conferring Mutations in Plasmodium falciparum Dihydrofolate Reductase: Polymerase Chain Reaction Methods for Surveillance in Africa. *The American Journal of Tropical Medicine and Hygiene*, **52**, 565–568.

3. Hofmann,N., Mwingira,F., Shekalaghe,S., Robinson,L.J., Mueller,I. and Felger,I. (2015) Ultra-Sensitive Detection of Plasmodium falciparum by Amplification of Multi-Copy Subtelomeric Targets. *PLOS Medicine*, **12**, e1001788.

4. Sundararaman,S.A., Plenderleith,L.J., Liu,W., Loy,D.E., Learn,G.H., Li,Y., Shaw,K.S., Ayouba,A., Peeters,M., Speede,S., *et al.* (2016) Genomes of cryptic chimpanzee Plasmodium species reveal key evolutionary events leading to human malaria. *Nature Communications*, **7**, 11078.

5. Oyola,S.O., Ariani,C.V., Hamilton,W.L., Kekre,M., Amenga-Etego,L.N., Ghansah,A., Rutledge,G.G., Redmond,S., Manske,M., Jyothi,D., *et al.* (2016) Whole genome sequencing of Plasmodium falciparum from dried blood spots using selective whole genome amplification. *Malaria Journal*, **15**, 597.

6. Gu,W., Crawford,E.D., O’Donovan,B.D., Wilson,M.R., Chow,E.D., Retallack,H. and DeRisi,J.L. (2016) Depletion of Abundant Sequences by Hybridization (DASH): using Cas9 to remove unwanted high-abundance species in sequencing libraries and molecular counting applications. *Genome Biology*, **17**, 1–13.

7. Wu,X., Scott,D.A., Kriz,A.J., Chiu,A.C., Hsu,P.D., Dadon,D.B., Cheng,A.W., Trevino,A.E., Konermann,S., Chen,S., *et al.* (2014) Genome-wide binding of the CRISPR endonuclease Cas9 in mammalian cells. *Nature Biotechnology*, **32**, 670–676.

8. Boyle,E.A., Andreasson,J.O.L., Chircus,L.M., Sternberg,S.H., Wu,M.J., Guegler,C.K., Doudna,J.A. and Greenleaf,W.J. (2017) High-throughput biochemical profiling reveals sequence determinants of dCas9 off-target binding and unbinding. *Proceedings of the National Academy of Sciences*, **114**, 5461–5466.

9. Gurobi Optimization, LLC (2018) Gurobi Optimizer Reference Manual.

10. Ruby,J.G., Bellare,P. and DeRisi,J.L. (2013) PRICE: software for the targeted assembly of components of (meta) genomic sequence data. *G3 Genes Genomes Genetics*, **3**.

11. Li,H., Handsaker,B., Wysoker,A., Fennell,T., Ruan,J. and Homer,N. (2009) The Sequence Alignment/Map format and SAMtools. *Bioinforma Oxf Engl.*, **25**.

12. Kearse,M., Moir,R., Wilson,A., Stones-Havas,S., Cheung,M., Sturrock,S., Buxton,S., Cooper,A., Markowitz,S., Duran,C., *et al.* (2012) Geneious Basic: an integrated and extendable desktop software platform for the organization and analysis of sequence data. *Bioinformatics*, **28**, 1647–1649.

13. Aurrecoechea,C., Brestelli,J., Brunk,B.P., Dommer,J., Fischer,S., Gajria,B., Gao,X., Gingle,A., Grant,G., Harb,O.S., *et al.* (2009) PlasmoDB: a functional genomic database for malaria parasites. *Nucleic Acids Res.*, **37**, D539-543.

14. Quinlan - 2014 - BEDTools The Swiss-Army Tool for Genome Feature A.pdf.

15. Hathaway,N.J., Parobek,C.M., Juliano,J.J. and Bailey,J.A. (2018) SeekDeep: single-base resolution de novo clustering for amplicon deep sequencing. *Nucleic Acids Res*, **46**, e21–e21.

Figure S1. Top: Antimicrobial resistance (AMR) gene guide RNA set design strategy. Bottom: Histograms showing properties of the 5,513 guide RNA set designed to target all known bacterial AMR genes.

Figure S2. For each FLASH-NGS sample, a sequencing depth between 500 and 5,000 reads was sufficient to recover 10 or more reads per gene for 100% of targeted genes. For NGS alone, at least 100-fold higher sequencing depth was required to achieve this threshold.

Figure S3. An extended set of 2,226 AMR-targeted guide RNAs was constructed and used in FLASH-NGS of isolate 1. Reads were filtered and aligned to the extended set of genes. One additional gene not targeted in the pilot guide RNA, blaZ-35, was detected and enriched with FLASH (see Table S5).

Figure S4. Varying amounts of Cas9 was used in FLASH-NGS of isolate 1. Each data point represents a single replicate.

Figure S5. Fraction of on-target reads in NGS and FLASH-NGS experiments on metagenomic patient samples.

Figure S6. Forty-eight *P. falciparum* genomic loci were targeted with FLASH-NGS.

Figure S7. Fraction of on-target reads in NGS and FLASH-NGS experiments on *P. falciparum* lab strain mixture samples.


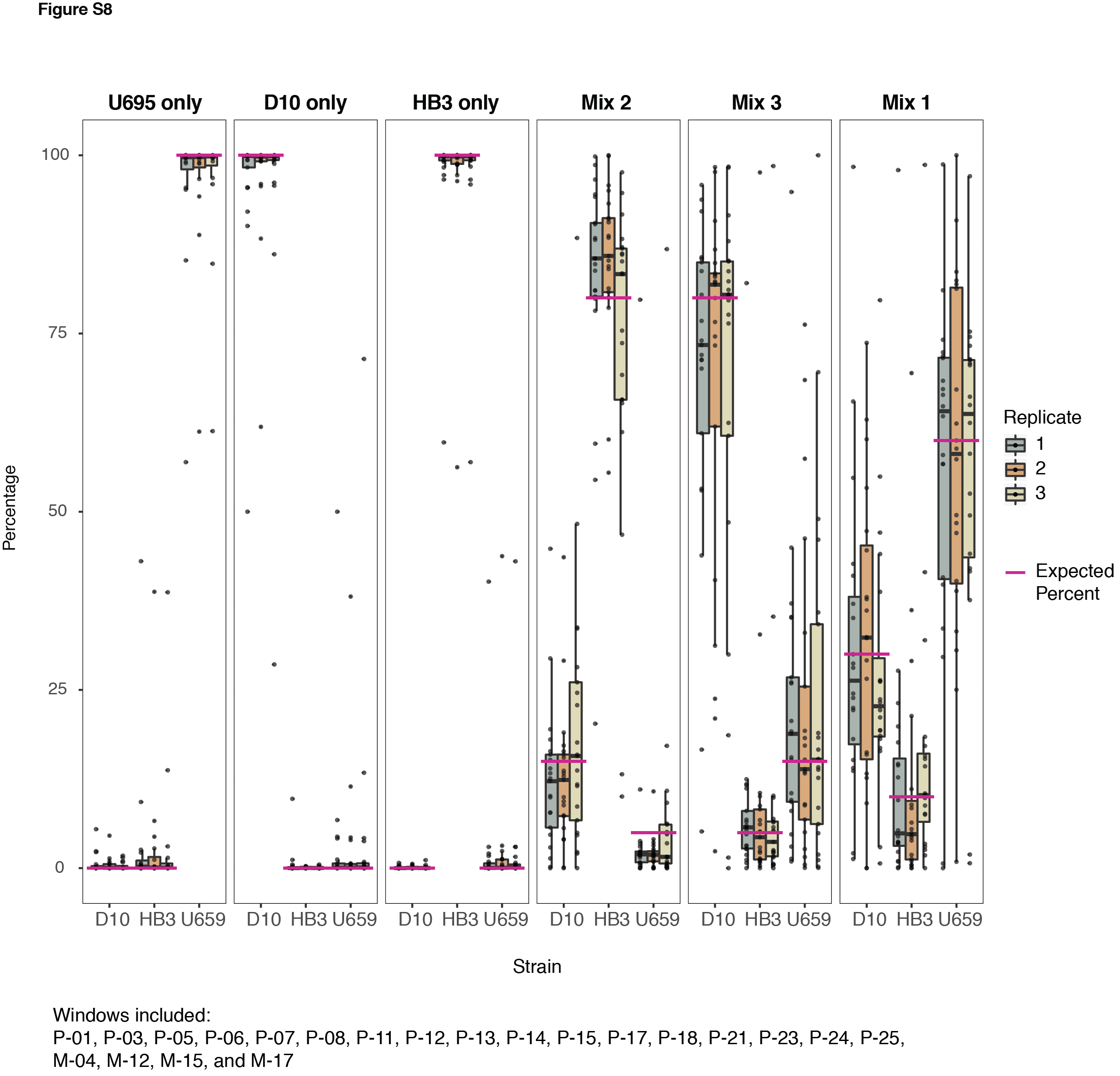


Figure S8. Three lab strains and three lab strain mixtures (see Fig. 4) were subjected to FLASH-NGS. For 21 of the 48 target windows, haplotype sequences uniquely identify each strain. Thus, for those windows, the observed percentages of reads belonging to each strain are plotted. Dots represent individual target windows. Boxes represent median and interquartile range. Pink lines indicate expected strain percentages. Windows included are: P-01, P-03, P-05, P-06, P-07, P-08, P-11, P-12, P-13, P-14, P-15, P-17, P-18, P-21, P-23, P-24, P-25, M-04, M-12, M-15, and M-17.

Figure S9. SeekDeep output indicating varying haplotype sequences for five patients infected with *P. falciparum*. Patients 5 and 6 have up to four haplotypes represented in each window; patients 7, 8 and 9 have up to three. Some windows had identical sequences across multiple patients, but none of these patients shared a complete set of identical primary haplotype sequences, suggesting that five different strains accounted for the primary infections in these five individuals. Haplotypes were colored as follows: First, primary haplotype sequences in patient 5, plus any identical sequences in the other patients, were colored light blue. Then primary haplotype sequences in patient 6, plus any identical sequences in other patients, were colored light purple. This was repeated for patients 7 (pink), 8 (light orange) and nine (light green). Next, secondary haplotype sequences in patient 5, plus any identical sequences in the other patients, were colored dark blue. This was repeated for secondary haplotypes in the remaining patients, then for tertiary haplotypes, and finally for quaternary haplotypes.

Table S6. Phenotypic drug resistance data for bacterial isolates involved in this study.
